# Diagnostic performance of dual-energy CT with electron-density reconstruction for lumbar disc herniation

**DOI:** 10.4102/sajr.v28i1.3000

**Published:** 2024-11-18

**Authors:** Umamaheshwari K. Basavaraju, Sushmita Balol, Vittal Manohar, Yashwanth Naik

**Affiliations:** 1 Department of Radiodiagnosis, Mysore Medical College and Research Institute, Mysore, India

**Keywords:** dual-energy computed tomography, electron-density reconstruction, magnetic resonance imaging, lumbar disc, intervertebral disc herniation, spinal nerve root impingement, standard computed tomography, retrospective study

## Abstract

**Background:**

Magnetic resonance imaging (MRI) is used for the evaluation of degenerative spinal disease. However, its utility is restricted in routine practice because of contraindications and a lack of widespread availability. Dual-energy computed tomography (DECT) is a newer technique for the evaluation of degenerative spinal disease.

**Objectives:**

This study aimed to evaluate the diagnostic performance of DECT with electron-density (ED) image reconstruction compared to standard CT for the detection of lumbar disc herniation, with MRI as the gold standard.

**Method:**

The retrospective study included 84 patients between 01 July 2023 to 31 December 2023 who underwent DECT and 1.5-T MRI within 1 week. Four radiologists, blinded to the clinical and MRI information, independently evaluated the standard CT series and DECT series with ED reconstructions for lumbar disc herniation and spinal nerve root impingement. The gold standard for comparison was lumbar spine MRI, and diagnostic accuracy was measured with sensitivity and specificity.

**Results:**

MRI revealed 417 lumbar disc herniations. Dual-energy computed tomography with ED reconstruction showed higher sensitivity (86.36% [532/616] vs. 57.79% [356/616]) and specificity (96.86% [1019/1052] vs. 95.82% [1008/1052]) for the detection of lumbar disc herniation compared to standard CT.

**Conclusion:**

Dual-energy computed tomography with ED reconstruction shows better diagnostic performance for the detection of lumbar disc herniation compared to standard CT and can be a useful alternative imaging modality when MRI is contraindicated or unavailable.

**Contribution:**

This study shows the usefulness of DECT as an alternative imaging technique for screening of degenerative spinal disease whenever MRI is contraindicated or unavailable.

## Introduction

The intervertebral disc is a cartilaginous structure comprising the annulus fibrosus (AF) and nucleus pulposus (NP). At the core of the disc is the NP, a gel-like material made of water and proteoglycans, held together by randomly arranged collagen fibres. The NP is surrounded by the ring-shaped AF, which is made up of 15–25 stacked sheets of collagen. Intervertebral disc disease is characterised by a decrease in proteoglycan content, resulting in dehydration in the NP, leading to morphological abnormalities and variations in its biomechanical properties.^1^ Herniated lumbar discs are a common degenerative condition associated with lower back pain with a large social and financial impact. Irreparable morbidity may result from complications related to spinal cord or spinal nerve root compression. Prompt and accurate diagnosis is essential for initiation of the best course of treatment and prevention of negative consequences.

Unenhanced lumbar spine CT is the principal modality to evaluate bone abnormalities such as fractures or any bone disorders because of its superiority over MRI for osseous detail.^2^ An MRI is the preferred modality for evaluating disc pathology, but its routine use is restricted by limited availability and patient contraindications. Imaging with standard CT is faster than MRI, with fewer contraindications, however, because of poor soft tissue contrast compared to MRI, standard CT has a low sensitivity and specificity for detecting herniated discs.^3,4^

Recent studies have endeavoured to identify disc herniation through the use of advanced CT imaging techniques such as Dual-energy computed tomography (DECT).^3,5,6,7,8^ Notably, recent publications have highlighted the value of three-material decomposition-based virtual non-calcium (VNCa) imaging for determining the presence of bone marrow oedema and identifying collagenous structures such as ligaments and tendons.^9,10,11^ Colour-coded VNCa imaging, in particular, has been found to be useful for the detection of disc pathology as observed by Booz et al.^3^

The Hounsfield unit (HU) value of each pixel on CT images is converted to create electron-density (ED) images, and the applied radiation dose is determined using a calibration curve specific to the scanner. As a calibration-free technique, single-source dual-layer DECT currently provides a more accurate ED map without conversion to HU. It permits ED imaging by distinguishing between Compton scattering and photoelectric effect attenuation, and measuring attenuation at two separate energies. The material density mostly affects Compton scattering, but in the high-energy range – such as during radiation therapy – the ED depends on Compton scattering. Although ED maps are frequently used to calculate radiation doses in radiation oncology, diagnostic radiology has not made much use of this technology.^3,12^

Shim et al., in a cross-sectional study on 64 patients (336 intervertebral discs), showed DECT with ED reconstruction can improve cervical disc herniation detection and diagnostic confidence compared with standard CT and VNCa images.^12^ To the authors’ knowledge, no studies have evaluated this ED post-processing technique on lumbar disc pathology. The aim of this study was therefore to compare the diagnostic performance and confidence of DECT with ED reconstruction to standard CT images for the purpose of detecting lumbar disc herniation and spinal nerve root impingement, using T2-weighted MRI as the reference standard. The novel ED post-processing approach was tested with colour-coded reconstruction on the premise that it may further improve the depiction of lumbar disc herniation.

## Research methods and design

**FIGURE 1:**
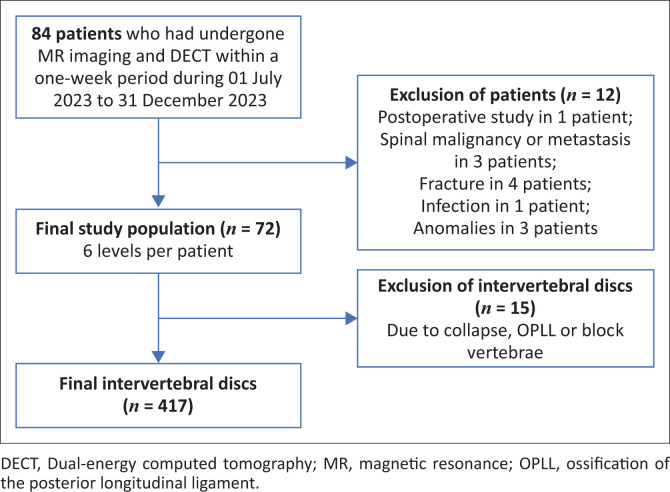
Flowchart showing selection of study population.

A retrospective study was conducted at the Department of Radiodiagnosis, Krishna Rajendra Hospital, Mysore Medical College and Research Institute in Mysore, India. All patients with low backache who underwent MRI and DECT within a 1-week period from 01 July 2023 to 31 December 2023 were included. Patients with a history of previous surgery (with metallic implants or intervertebral disc surgeries), spinal tumours or infections, a history of trauma and vertebral anomalies were excluded. The study population selection is presented in Figure 1.

### CT protocol

Non-contrast CT scans were acquired using a single-source, 128-slice dual-energy CT system (Somatom definition edge; Siemens Healthineers, Erlangen, Germany). By utilising a Sn (tin filter) and Au (gold filter), the X-ray tube was operated at 120 kV and 440 mAs. A dual-energy protocol (rotation time: 0.33 s; pitch factor: 0.35; collimation: 38 mm × 0.6 mm) was used to perform CT in the craniocaudal direction. Mean volume CT dose index was 11.683 mGy (range, 9.730 mGy – 14.060 mGy) and mean dose-length product was 478.099 mGy.cm (range, 350.770 mGy.cm – 691.900 mGy.cm).

Three separate sets of images were obtained in each dual-energy CT scan: Au120 kVp, Sn120 kVp, and weighted average (ratio, 0.5:0.5) to resemble the contrast provided by a single-energy 120-kVp image. This was followed by generation of axial and sagittal reconstructions (1 mm section thickness) with a specific dual-energy medium-soft convolution kernel. Post-processing was performed using commercially available universal imaging software (syngo.via, version VB60A_HF02; Siemens Healthineers, Erlangen, Germany). An ED reconstruction algorithm optimised for analysis of intervertebral discs was applied by using dedicated software settings (colour lookup tables low-energy value, spectrum; colour lookup tables high-energy value, grayscale; CT preset 1-liver). The DECT axial and sagittal ED reconstructions and standard CT series were used for image analysis. The readers were allowed to freely change the window width and level during the image assessment in order to improve the detection of disc herniation.

### Magnetic resonance imaging protocol

All patients underwent non-contrast MRI with a 1.5-T system (uMR 570; 24 channel United Imaging, Jiading, Shanghai, China) using a dedicated spine surface coil. Standard T1-weighted fast spin-echo (repetition time msec/echo time msec, 615/8.3; matrix size, 256/0/0/192; slice thickness, 3 mm), T2-weighted fast spin-echo (2000/80.6; matrix size, 304/0/0/243; slice thickness, 3 mm) sagittal sequences of the lumbosacral spine were performed. The T2-weighted fast spin-echo (repetition time msec/echo time msec, 5095/111.8; matrix size, 288/0/0/184; slice thickness, 3mm) was performed in the axial plane.

### Image analysis

Image evaluation was performed with syngo.via, version VB60A_HF02 (Siemens Healthineers, Erlangen, Germany). Initially, to establish the reference standard, two medical council-certified radiologists, blinded to the clinical and CT information, evaluated the MRI scans in consensus, to detect the type and presence of disc herniation (according to the lumbar disc pathologic classification of the North American Spine Society version 2.0),^12,13^ and the presence of lumbar disc herniation related spinal nerve root impingement. Lumbar disc herniation is defined as a focal displacement (< 25% of the disc circumference) of disc material beyond the limits of the intervertebral disc space. Lumbar disc herniation was classified as protrusion (distance between the edges of the disc herniation is less than the distance between the edges of the base), extrusion (distance between the edges of the herniated disc material is greater than the distance at the base in at least one plane), or sequestration (displaced disc material has lost continuity with the parent disc) by each reader.^3,13^ The base is defined as the width of disc material at the outer margin of the disc space of origin, where disc material displaced beyond the disc space is continuous with disc material within the disc space.^3,13^

Bulging lumbar disc is generalised extension (> 25% of the disc circumference) of disc tissue beyond the edges of the ring apophyses and considered as non-herniated discs according to the lumbar disc pathologic classification of the North American Spine Society.^13^ Each reader was tasked to record every spinal nerve root impingement related to bulging or herniating lumbar discs. The readers were free to adjust the window settings and scroll through the entire stack of MRI data. The five-point Likert scale (1, unacceptable; 5, excellent) was used to assess the image quality and diagnostic confidence.

After establishing the reference standard, four radiologists experienced in musculoskeletal imaging and blinded to the MRI results and clinical information, were tasked to independently assess the DECT and standard CT images. Initially, grayscale standard CT series were presented to the readers in random order, to assess and record the presence and type of lumbar disc herniation and spinal nerve root impingement on a per-disc basis and on a per-patient basis. After a 6–8-week interval, to avoid potential recall bias, readers were presented with DECT images in a random order and tasked to independently evaluate the colour-coded ED reconstructions without having access to the standard CT data. Each reader was allowed to freely adjust the window settings and scroll through the entire CT series. The five-point Likert scales were used to assess image quality and diagnostic confidence across all CT series.^5^

### Statistical analysis

Microsoft Excel (Redmond, Washington, United States), IBM SPSS (Armonk, New York, United States), MedCalc Software Ltd. (Ostend, Belgium). Data were entered in an Excel spreadsheet. Statistical analysis was performed using SPSS (Statistical Package for Social Sciences) version 20 (IBM SPASS statistics [IBM corp. released 2011]), MedCalc Software Ltd (Diagnostic test evaluation calculator, Version 22.023). Descriptive statistics of the variables was calculated through means and standard deviations for quantitative variables, frequency and proportions for qualitative variables. Chi-square test was applied to find the association of qualitative variables. The ROC curve was used to find the cut-off values, sensitivity and specificity of ED dual energy CT to predict lumbar disc herniation. A *p* < 0.05 was considered to indicate statistical significance. The standard *t*-test was applied to determine statistical significance of quantitative data. On a per-disc and per-patient basis, sensitivity, specificity, positive predictive values (PPVs), negative predictive values (NPVs) and accuracy values were computed. Clustering of intervertebral discs per patient following the method described by Genders et al.^14^ for each reader and consensus reading on the basis of a contingency table was taken into account. Logistic regression analysis with a robust variance estimator was used.^3^ In order to account for intervertebral disc clustering per patient, generalised estimating equations with an independent correlation working matrix and robust variance estimator were used to assess the sensitivity, specificity, PPV and NPV of the two CT techniques.^3^

Inter-reader agreement was evaluated by computing weighted Fleiss k. Kappa results were qualitatively stratified by score κ = 0.81–1.00, almost perfect agreement; κ = 0.61–0.80, substantial agreement; κ = 0.41–0.60, moderate agreement; κ = 0.21–0.40, fair agreement; and κ < 0.20, slight agreement.^15^ All values of overall sensitivity, specificity, PPV, NPV and accuracy are expressed as means with 95% confidence intervals.

### Ethical considerations

This retrospective study was approved by the Institutional Ethics Committee, Mysore medical college and Research Institute and associated hospitals. Ethical committee clearance was obtained on 18 January 2024. Patient consent for individual cases was waived as all studies were retrospectively collected from the institutional Picture Archiving and Communication System (PACS) and studies were anonymised prior to review by readers.^16^

## Results

A total of 417 lumbar intervertebral discs (median per patient, six) in 72 patients (mean age, 49.7 years; range, 38–69 years) were included. A total of 39 males (54.2%; mean age, 49.8 years; range, 38–69 years) and 33 females (45.8%; mean age, 49.5 years; range, 39–66 years) were included. MRI revealed 154 lumbar herniated discs (36.9% of all intervertebral discs) and 96 instances of spinal nerve root impingement. There were 130 protrusions (31.1%), 19 extrusions (4.6%) and three sequestrations (1.2%). The mean examination interval between dual energy CT and MRI was 3 days (range, 0–5 days).

### Diagnostic accuracy per intervertebral disc

Colour-coded ED reconstructions showed higher overall sensitivity, specificity, PPV, NPV and accuracy for the detection of lumbar disc herniation compared with standard CT, taking clustering into account.^3^ The sensitivity, NPV and accuracy data were statistically significant for all readers (Table 1). Inter reader agreement was almost perfect for ED images (*k* = 0.99) and standard CT (*k* = 0.90, *p* < 0.0001).

**TABLE 1:** Comparison of diagnostic performance of electron-density reconstruction and Standard CT for each reader for the detection of lumbar disc herniation per intervertebral disc.

Parameter	Sensitivity (%)					Specificity (%)					PPV (%)					NPV (%)					Accuracy (%)				
	*n*	*N*	%	95% CI	*p*	*n*	*N*	%	95% CI	*p*	*n*	*N*	%	95% CI	*p*	*n*	*N*	%	95% CI	*p*	*n*	*N*	%	95% CI	*p*
**Average**	-	-	-	-	<0.0001*	-	-	-	-	0.222	-	-	-	-	0.0036*	-	-	-	-	<0.0001*	-	-	-	-	<0.0001*
Standard CT	356	616	57.79	53.78–61.73	-	1008	1052	95.82	94.43–96.94	-	356	400	89.00	85.74–91.59	-	1008	1268	79.50	77.93–80.97	-	1364	1668	81.77	79.84–83.60	-
ED	532	616	86.36	83.40–88.98	-	1019	1052	96.86	95.62 – 97.83	-	532	565	94.16	92.00 – 95.76	-	1019	1103	92.38	90.86 – 93.67	-	1551	1668	92.99	91.65 – 94.16	-
**Reader 1**	-	-	-	-	< 0.0001*	-	-	-	-	0.66	-	-	-	-	0.196	-	-	-	-	< 0.0001*	-	-	-	-	<0.0001*
Standard CT	86	154	55.84	47.63 – 63.83	-	253	263	96.20	93.12 – 98.16	-	86	96	89.58	82.17 – 94.13	-	253	321	78.82	75.67 – 81.65	-	339	417	81.29	77.21 – 84.92	-
ED	132	154	85.71	79.17 – 90.83	-	255	263	96.96	94.09 – 98.68	-	132	140	94.29	89.26 – 97.04	-	255	277	92.06	88.72 – 94.47	-	387	417	92.81	89.89 – 95.09	-
**Reader 2**	-	-	-	-	< 0.0001*	-	-	-	-	0.167	-	-	-	-	0.027*	-	-	-	-	< 0.0001*	-	-	-	-	< 0.0001*
Standard CT	94	154	61.04	52.86 – 68.78	-	248	263	94.30	90.77 – 96.77	-	94	109	86.24	79.05 – 91.23	-	248	308	80.52	77.19 – 83.47	-	342	417	82.01	77.98 – 85.58	-
ED	134	154	87.01	80.66 – 91.88	-	255	263	96.96	94.09 – 98.68	-	134	142	94.37	89.41 – 97.08	-	255	275	92.73	89.44 – 95.05	-	389	417	93.29	90.44 – 95.49	-
**Reader 3**	-	-	-	-	< 0.0001*	-	-	-	-	0.67	-	-	-	-	0.0015*	-	-	-	-	< 0.0001*	-	-	-	-	< 0.0001*
Standard CT	93	154	60.39	52.20 – 68.17	-	252	263	95.82	92.64 – 97.89	-	93	104	89.42	82.38 – 93.86	-	252	313	80.51	77.24 – 83.41	-	345	417	82.73	78.76 – 86.24	-
ED	134	154	87.01	80.66 – 91.88	-	254	263	96.58	93.60 – 98.42	-	134	143	93.71	88.65 – 96.60	-	254	274	92.70	89.40 – 95.03	-	388	417	93.05	90.16 – 95.29	-
**Reader 4**	-	-	-	-	< 0.0001*	-	-	-	-	0.4	-	-	-	-	0.089	-	-	-	-	< 0.0001*	-	-	-	-	< 0.0001*
Standard CT	88	154	57.14	48.93 – 65.08	-	251	263	95.44	92.17 – 97.62	-	88	100	88.00	80.58 – 92.84	-	251	317	79.18	75.98 – 82.05	-	339	417	81.29	77.21 – 84.92	-
ED	132	154	85.71	79.17 – 90.83	-	255	263	96.96	94.09 – 98.68	-	132	140	94.29	89.26 – 97.04	-	255	277	92.06	88.72 – 94.47	-	387	417	92.81	89.89 – 95.09	-

Note: *n*- Numerator; *N*- Denominator; % = *n/N* × 100.

CI, confidence interval; CT, computed tomography; ED, electron-density; NPV, negative predictive value; PPV, positive predictive value.

* , *p* < 0.05 is significant.

**FIGURE 2:**
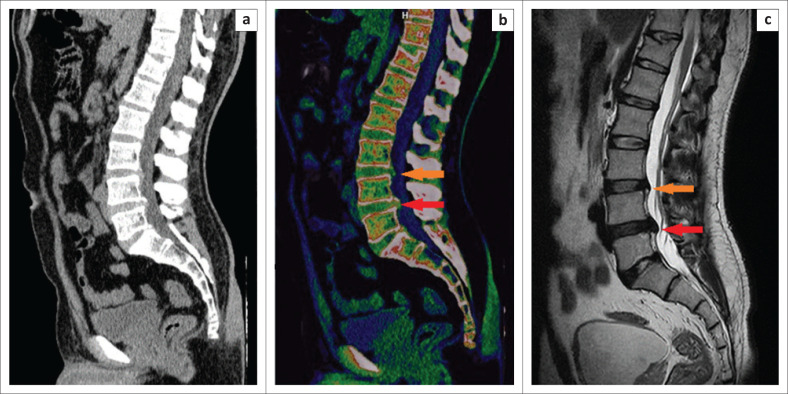
A 40-year-old man who presented with chronic lower back pain with recent aggravation: (a) The lumbar disc protrusions at L3/L4 and L4/L5 levels were initially not clearly depicted on the sagittal grayscale standard CT reconstructions. (b) The lumbar disc protrusions (orange and red arrows) depicted on the sagittal colour-coded electron-density reconstruction images optimised for analysis of intervertebral discs. (c) Non-contrast sagittal T2-weighted MRI confirmed the diagnosis.

Statistical analysis revealed higher overall sensitivity, specificity, PPV, NPV and accuracy for the detection of lumbar disc protrusion by using ED reconstructions compared with standard CT, taking clustering into account, with statistically significant correlations for sensitivity, NPV and accuracy (Table 2) (Figure 2). Inter reader agreement was almost perfect for ED images (*k* = 0.99) and for standard CT (*k* = 0.87, *p* < 0.0001).

**TABLE 2:** Comparison of diagnostic performance of electron-density reconstruction and Standard CT for the detection of type of lumbar disc herniation and spinal nerve root impingement per intervertebral disc.

Parameter	Sensitivity (%)					Specificity (%)					PPV (%)					NPV (%)					Accuracy (%)				
	*n*	*N*	%	95% CI	*p*	*n*	*N*	%	95% CI	*p*	*n*	*N*	%	95% CI	*p*	*n*	*N*	%	95% CI	*p*	*n*	*N*	%	95% CI	*p*
**Lumbar disc protrusion**	-	-	-	-	< 0.001*	-	-	-	-	0.021*	-	-	-	-	< 0.001*	-	-	-	-	< 0.001*	-	-	-	-	< 0.001*
Standard CT	273	520	52.50	48.11–56.86	-	1094	1148	95.30	93.91–96.45	-	273	327	83.49	79.37–86.91	-	1094	1341	81.58	80.17–82.91	-	1367	1668	81.95	80.02–83.77	-
ED	438	520	84.23	80.81–87.26	-	1116	1148	97.21	96.09–98.09	-	438	470	93.19	90.66–95.07	-	1116	1198	93.16	91.77–94.32	-	1554	1668	93.17	91.85–94.33	-
**Lumbar disc extrusion**	-	-	-	-	< 0.001*	-	-	-	-	0.38	-	-	-	-	0.22	-	-	-	-	< 0.001*	-	-	-	-	< 0.001*
Standard CT	57	76	75.00	63.74–84.23	-	1586	1592	99.62	99.18–99.86	-	57	63	90.48	80.88–95.52	-	1586	1605	98.82	98.26–99.19	-	1643	1668	98.50	97.80–99.03	-
ED	73	76	96.05	88.89–99.18	-	1589	1592	99.81	99.45–99.96	-	73	76	96.05	88.70–98.69	-	1589	1592	99.81	99.43–99.94	-	1662	1668	99.64	99.22–99.87	-
**Lumbar disc sequestration**	-	-	-	-	< 0.001*	-	-	-	-	0.56	-	-	-	-	0.41	-	-	-	-	0.0015*	-	-	-	-	0.012*
Standard CT	9	20	45.00	23.06–68.47	-	1647	1648	99.94	99.66–100.00	-	9	10	90.00	54.46–98.55	-	1647	1658	99.34	99.02–99.55	-	1656	1668	99.28	98.75–99.63	-
ED	18	20	90.00	68.30–98.77	-	1648	1648	100.00	99.78–100.00	-	18	18	100.00	81.47–100.00	-	1648	1650	99.88	99.55–99.97	-	1666	1668	99.88	99.57–99.99	-
**Spinal nerve root impingement**	-	-	-	-	< 0.001*	-	-	-	-	0.176	-	-	-	-	< 0.001*	-	-	-	-	< 0.001*	-	-	-	-	< 0.001*
Standard CT	173	384	45.05	40.00–50.18	-	1240	1284	96.57	95.43–97.50	-	173	217	79.72	74.24–84.29	-	1240	1451	85.46	84.29–86.55	-	1413	1668	84.71	82.89–86.41	-
ED	305	384	79.43	75.03–83.36	-	1252	1284	97.51	96.50–98.29	-	305	337	90.50	87.09–93.09	-	1252	1331	94.06	92.87–95.07	-	1557	1668	93.35	92.04–94.49	-

Note: *n*- Numerator; *N*- Denominator; % = *n/N* × 100.

CI, confidence interval; CT, computed tomography; ED, electron-density; NPV, negative predictive value; PPV, positive predictive value.

* , *p* < 0.05 is significant.

**FIGURE 3:**
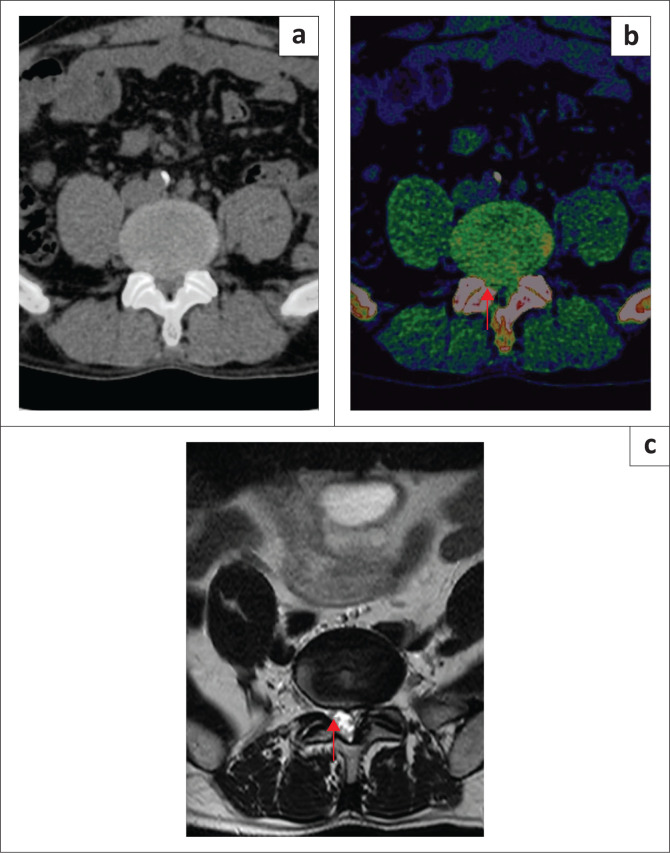
A 58-year-old man who presented with lower back pain and paraesthesia in the right lower limb: (a) Bulging L5/S1 intervertebral disc with impingement of the traversing right S1 nerve root was underestimated on standard grayscale axial CT. (b) Diagnosis of spinal nerve root impingement was made by all readers on the axial colour-coded electron-density reconstruction images. (c) Spinal nerve root impingement was confirmed on axial T2-weighted MRI (red arrow).

**FIGURE 4:**
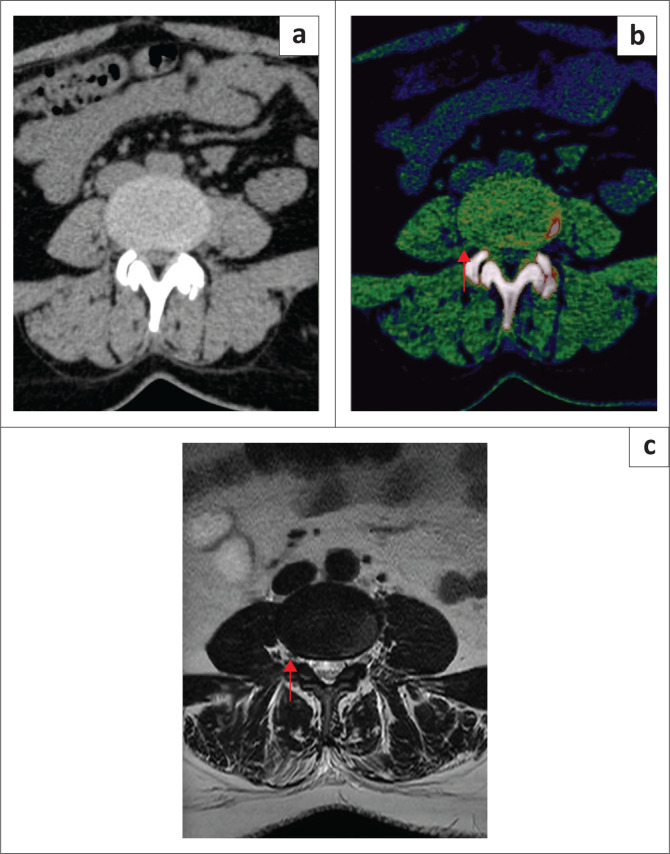
A 53-year-old woman who presented with lower back pain: (a) Bulging L4/L5 intervertebral disc with nerve root impingement of the exiting right L4 nerve root was underestimated on standard grayscale axial CT images. (b) Diagnosis of spinal nerve root impingement was made by all readers on the axial colour-coded electron-density reconstruction images. (c) Spinal nerve root impingement was confirmed on axial T2-weighted MRI (red arrow).

For the detection of spinal nerve root impingement, higher overall sensitivity, specificity, PPV, NPV and accuracy were noticed when comparing DECT ED reconstructions with standard CT, taking clustering into account (Table 3) (Figure 3 and Figure 4). The *p*-values were < 0.05 for sensitivity, NPV and accuracy for all readers. Inter reader agreement was almost perfect for ED images (*k* = 1) and substantial for standard CT (*k* = 0.79).

**TABLE 3:** Comparison of diagnostic performance of electron-density reconstruction and Standard CT for each reader for the detection of spinal nerve root impingement per intervertebral disc.

Parameter	Sensitivity (%)					Specificity (%)					PPV (%)					NPV (%)					Accuracy (%)				
	*n*	*N*	%	95% CI	*p*	*n*	*N*	%	95% CI	*p*	*n*	*N*	%	95% CI	*p*	*n*	*N*	%	95% CI	*p*	*n*	*N*	%	95% CI	*p*
**Average**	-	-	-	-	< 0.001*	-	-	-	-	0.176	-	-	-	-	< 0.001*	-	-	-	-	< 0.001*	-	-	-	-	< 0.001*
**Standard CT**	173	384	45.05	40.00–50.18	-	1240	1284	96.57	95.43–97.50	-	173	217	79.72	74.24–84.29	-	1240	1451	85.46	84.29–86.55	-	1413	1668	84.71	82.89–86.41	-
**ED**	305	384	79.43	75.03–83.36	-	1252	1284	97.51	96.50–98.29	-	305	337	90.50	87.09–93.09	-	1252	1331	94.06	92.87–95.07	-	1557	1668	93.35	92.04–94.49	-
**Reader 1**	-	-	-	-	< 0.001*	-	-	-	-	0.661	-	-	-	-	0.118	-	-	-	-	< 0.001*	-	-	-	-	< 0.001*
**Standard CT**	43	96	44.79	34.63–55.29	-	311	321	96.88	94.35–98.50	-	43	53	81.13	69.20–89.17	-	311	364	85.44	83.04–87.55	-	354	417	84.89	81.09–88.19	-
**ED**	76	96	79.17	69.67–86.79	-	313	321	97.51	95.15–98.92	-	76	84	90.48	82.63–94.99	-	313	333	93.99	91.37–95.85	-	389	417	93.29	90.44–95.49	-
**Reader 2**	-	-	-	-	< 0.001*					0.307					0.021*					< 0.001*					< 0.001*
**Standard CT**	42	96	43.75	33.64–54.25	-	308	321	95.95	93.17–97.83	-	42	55	76.36	64.43–85.21	-	308	362	85.08	82.68–87.20	-	350	417	83.93	80.05–87.33	-
**ED**	77	96	80.21	70.83–87.64	-	313	321	97.51	95.15–98.92	-	77	85	90.59	82.82–95.05	-	313	332	94.28	91.67–96.10	-	390	417	93.53	90.72–95.69	-
**Reader 3**	-	-	-	-	< 0.001*	-	-	-	-	0.66	-	-	-	-	0.14	-	-	-	-	< 0.001*	-	-	-	-	< 0.001*
**Standard CT**	45	96	46.88	36.61–57.34	-	311	321	96.88	94.35–98.50	-	45	55	81.82	70.22–89.57	-	311	362	85.91	83.47–88.05	-	356	417	85.37	81.61–88.62	-
**ED**	76	96	79.17	69.67–86.79	-	313	321	97.51	95.15–98.92	-	76	84	90.48	82.63–94.99	-	313	333	333	91.37–95.85	-	389	417	93.29	90.44–95.49	-
**Reader 4**	-	-	-	-	< 0.001*	-	-	-	-	0.52	-	-	-	-	0.072	-	-	-	-	< 0.001*	-	-	-	-	< 0.001*
**Standard CT**	43	96	44.79	34.63–55.29	-	310	321	96.57	93.95–98.28	-	43	54	79.63	67.73–87.92	-	310	363	85.40	82.99–87.52	-	353	417	84.65	80.83–87.98	-
**ED**	76	96	79.17	69.67–86.79	-	313	321	97.51	95.15–98.92	-	76	84	90.48	82.63–94.99	-	313	333	93.99	91.37–95.85	-	389	417	93.29	90.44–95.49	-

Note: *n*- Numerator; *N*- Denominator; % = *n/N* × 100.

CI, confidence interval; CT, computed tomography; ED, electron-density; NPV, negative predictive value; PPV, positive predictive value.

* , *p* < 0.05 is significant.

### Diagnostic accuracy per patient

The analysis per patient of colour-coded ED reconstructions for the detection of lumbar disc protrusion compared with standard grayscale CT images showed higher overall sensitivity, specificity, PPV, NPV and accuracy, with *p* < 0.05 for sensitivity, NPV and accuracy. For the detection of spinal nerve root impingement, higher overall sensitivity, specificity, PPV, NPV and accuracy were noted with colour-coded ED reconstructions compared to standard CT (Table 4). The data for sensitivity, NPV and accuracy were statistically significant. Inter reader agreement for ED reconstructions and for standard CT images was almost perfect and substantial for the detection of lumbar disc protrusion (*k* = 1 vs. 0.71), lumbar disc extrusion (*k* = 0.93 vs. 0.73), lumbar disc sequestration (*k* = 0.88 vs. 0.65), and spinal nerve root impingement (*k* = 1 vs. 0.79). The *p*-values for all comparisons were < 0.0001.

**TABLE 4:** Comparison of Diagnostic performance of electron-density Reconstruction and Standard CT for the detection of type of lumbar disc herniation and spinal nerve root impingement per patient.

Parameter	Sensitivity (%)					Specificity (%)					PPV (%)					NPV (%)					Accuracy (%)				
	*n*	*N*	%	95% CI	*p*	*n*	*N*	%	95% CI	*p*	*n*	*N*	%	95% CI	*p*	*n*	*N*	%	95% CI	*p*	*n*	*N*	%	95% CI	*p*
**Lumbar disc protrusion**	-	-	-	-	0.0002*	-	-	-	-	0.17	-	-	-	-	0.242	-	-	-	-	0.0002*	-	-	-	-	0.0002*
Standard CT	246	280	87.86	83.45–91.44	-	6	8	75.00	34.91–96.81	-	246	248	99.19	97.37–99.76	-	6	40	15.00	9.59–22.70	-	252	288	87.50	83.12–91.09	-
ED	280	280	100	98.69–100.00	-	8	8	100.00	63.06–100.00	-	280	280	100	98.69–100.00	-	8	8	100	63.06–100.00	-	288	288	100.00	98.73–100.00	-
**Lumbar disc extrusion**	-	-	-	-	0.0024*	-	-	-	-	0.380	-	-	-	-	0.226	-	-	-	-	0.0044*	-	-	-	-	0.0022*
Standard CT	59	76	77.63	66.62–86.40	-	206	212	97.17	93.94–98.95	-	59	65	90.77	81.58–95.62	-	206	223	92.38	88.85–94.85	-	265	288	92.01	88.26–94.87	-
ED	73	76	96.05	88.89–99.18	-	209	212	98.58	95.92–99.71	-	73	76	96.05	88.77–98.68	-	209	212	98.58	95.83–99.53	-	282	288	97.92	95.52–99.23	-
**Lumbar disc sequestration**	-	-	-	-	0.0012	-	-	-	-	1.100	-	-	-	-	0.412	-	-	-	-	0.0014*	-	-	-	-	0.0144*
Standard CT	9	20	45.00	23.06–68.47	-	267	268	99.63	97.94–99.99	-	9	10	90.00	54.53–98.54	-	267	278	96.04	94.23–97.30	-	27	288	95.83	92.83–97.83	-
ED	18	20	90.00	68.30–98.77	-	268	268	100.00	98.63–100.00	-	18	18	100.00	81.47–100.00	-	268	270	99.26	97.30–99.80	-	286	288	99.31	97.51–99.92	-
**Spinal nerve root impingement**	-	-	-	-	< 0.0001*	-	-	-	-	0.092	-	-	-	-	0.106	-	-	-	-	< 0.0001*	-	-	-	-	< 0.0001*
Standard CT	180	276	65.22	59.28–70.83	-	9	12	75.00	42.81–94.51	-	180	183	98.36	95.73–99.38	-	9	105	8.57	6.11–11.89	-	189	288	65.62	59.83–71.10	-
ED	232	276	84.06	79.20–88.17	-	12	12	100.00	73.54–100.00	-	232	232	100.00	98.42–100.00	-	12	56	21.43	17.22–26.34	-	244	288	84.72	80.04–88.67	-

Note: *n*- Numerator; *N*- Denominator; % = *n/N* × 100.

CI, confidence interval; CT, computed tomography; ED, electron-density; NPV, negative predictive value; PPV, positive predictive value.

* , *p* < 0.05 is significant.

### Diagnostic confidence

The diagnostic confidence for lumbar disc herniation was very high for MRI (mean ~ 4.9), very high for DECT colour-coded ED reconstructions (mean ~ 4.8), and low for standard CT images (mean ~ 2.9). Inter reader agreement was good for ED images (*k* = 0.88), and for standard CT (*k* = 0.64).

## Discussion

The findings in this study indicated that colour-coded ED reconstructions were more accurate in diagnosing lumbar disc herniation, protrusion and spinal nerve root impingement than standard CT. Colour-coded ED reconstructions had substantially higher overall sensitivity than standard CT for demonstrating lumbar disc herniation, protrusion, extrusion, sequestration and nerve impingement. Colour-coded ED reconstructions showed high (≥ 90%) overall specificity, PPV, NPV and accuracy for the detection of lumbar disc herniation, protrusion, extrusion, sequestration and nerve root impingement. Higher values were observed for ED reconstruction in all categories in comparison with standard grayscale CT. In addition, colour-coded ED reconstructions had a higher inter-reader agreement for the detection of lumbar disc herniation and spinal nerve root impingement compared to standard CT.

When MRI is not available or may be contraindicated, dual-energy CT could be an excellent alternative imaging modality, as supported by the high diagnostic accuracy of ED reconstructions for the diagnosis of lumbar disc herniation and nerve root impingement in this study. This study results were consistent with a previous study by Shim et al. on cervical disc herniations, which showed a higher sensitivity (94% vs. 76%) and accuracy (93% vs. 80%), respectively, for colour-coded ED reconstructions compared to standard CT.^12^

Colour overlay in ED reconstructions created excellent contrast to differentiate intervertebral discs from bones and spinal canal contents. Consequently, readers were more accurate with the colour overlay ED maps than standard CT in detecting small herniations. A previous study by Shim et al. showed that colour-coded ED maps provided higher noise suppression and increased smoothness resulting in higher subjective image quality than VNCa images.^12^ Reconstructing colour-coded ED pictures from DECT took only 2 min on average; therefore, the reconstruction algorithm is appropriate for everyday clinical practice.

This study had several limitations. Firstly, only 72 patients were included because of the retrospective single-centre study design and a 1-week examination delay between dual-energy CT and MRI. Secondly, this study used MRI as the reference standard for the diagnosis of lumbar disc herniation. Earlier studies considered surgery as the standard of reference while assessing the diagnostic accuracy of standard CT for lumbar spine diseases,^5^ because MRI may exaggerate the degree of disc herniation and spinal nerve root impingement.^17,18^ Forristall et al. showed that MRI was 90.3% accurate in the evaluation of lumbar disc herniation, compared to surgical findings.^19^ Jackson et al. showed that MRI was 76.5% accurate in the detection of herniated NP, and had a false positive rate of 13.2% and false negative rate of 22.7%.^20^ As per a systematic review performed by Kim et al., MRI showed a sensitivity ranging from 64% to 93% and specificity ranging from 55% to 100%, with wide confidence intervals, compared to surgical findings.^21^ Thirdly, although DECT is available with multiple vendors, the findings are currently only applicable to the specific CT system and post-processing software used in this study. Fourthly, recall bias and statistical distortion could have resulted from the evaluation of standard grayscale CT and DECT ED reconstructions over the 6-8 week interval.

### Implications and recommendations

This study demonstrates the value of DECT as a substitute imaging modality for the detection of degenerative spinal disease in situations where MRI is either not available or contraindicated. However, multicentre research studies with higher sample sizes are indicated for further validation.

## Conclusion

This research demonstrated that compared to standard CT, the colour-coded DECT ED reconstruction technique produced significantly better diagnostic accuracy for the identification of lumbar disc herniation and spinal nerve root impingement when using MRI as the reference standard. Similar diagnostic confidence and image quality were noticed for ED and MR images. Therefore, colour-coded DECT ED reconstruction can offer an excellent alternative when MRI is unavailable or contraindicated.
